# Effects of Systemic Enzyme Supplements on Symptoms and Quality of Life in Patients with Pulmonary Fibrosis—A Pilot Study

**DOI:** 10.3390/medicines8110068

**Published:** 2021-11-05

**Authors:** Neha Shah

**Affiliations:** Pulmonary Fibrosis NOW, Chino, CA 91710, USA; drnehashah772@gmail.com

**Keywords:** pulmonary fibrosis, idiopathic pulmonary fibrosis, dietary supplements, systemic enzyme therapy, health related quality of life

## Abstract

Current FDA-approved antifibrotic treatments for Idiopathic Pulmonary Fibrosis slow down disease progression but have little impact on symptoms or quality of life in patients. This study was conducted to evaluate the effects of systemic enzymes in relieving symptoms associated with PF and improving quality of life. Methods: an open-label, prospective study on subjects with a confirmed diagnosis of PF was conducted as proof-of-concept. The subjects (*n* = 13) received the oral systemic enzyme supplements Serracor-NK and Serra Rx for 12 weeks and completed Health-Related Quality of Life (HRQL) questionnaires. The effect of this regimen was examined by comparing the end-of-treatment questionnaire scores with baseline values. Results: significant improvement was seen in 61.5% of subjects, as assessed by the WHO well-being index; an improvement in scores was seen in 84.6% of the subjects, as assessed by the UCSD Shortness of Breath Questionnaire, with 38.4% of the subjects showing minimal clinically important difference; the supplementation was found to be efficacious in 69.2%, 84.6%, 69.2% and 61.5% of the subjects, as assessed by the Saint George’s Respiratory Questionnaire total, symptom, activity, and impact scores, respectively. Conclusions: Serracor-NK and Serra Rx improve symptoms, as well as mental and physical wellbeing and HRQL in patients with PF.

## 1. Introduction

Idiopathic pulmonary fibrosis (IPF) is a progressive disease, with a high level of mortality during the first 3–5 years following diagnosis [1,2]. In pulmonary fibrosis (PF), there is a build-up of fibrin in the interstitial tissue of the lungs, which causes the lung tissue to thicken and become stiff. IPF is characterized by poor quality of life, mainly because of the burden of symptoms such as dyspnea and cough, usually occurring many months before diagnosis [3]. Progressive activity-limiting dyspnea is the main symptom of IPF and is a major concern of all PF patients and their caregivers. Patients also frequently experience fear, anxiety, worry, hopelessness and helplessness [4]. Due to its symptoms and their resultant impact on physical, social, and emotional well-being, patients with IPF suffer from decreased health-related quality of life [5,6].

The treatment options for IPF are very limited. Once developed, lung scarring is permanent and there is no evidence of any available medication that can reverse this condition. Although available antifibrotic therapies slow down disease progression, they have no impact on quality of life. These drugs have also been associated with side effects such as appetite loss, nausea, diarrhea, lethargy, and photosensitivity.

There is historical, anecdotal and some preclinical and clinical evidence on the use of serrapeptase and nattokinase for therapeutic applications, owing to their fibrinolytic, anti-inflammatory, and immunomodulatory effects. Supplementation with these enzymes may be beneficial in limiting and even reducing the amount of scar tissue, thus alleviating symptoms. The fibrinolytic enzymes serrapeptase and nattokinase have been shown to be effective in removing fibrous scar tissue [7,8]. Serratiopeptidase, commonly known as serrapeptase, is widely used in surgery, orthopedics, otorhinolaryngology, gynecology, and dentistry for its anti-inflammatory, anti-edemic and analgesic effects [9]. Further, serrapeptase is also known to have beneficial effects in patients with chronic airway diseases. Another proteolytic enzyme, nattokinase, demonstrated fibrinolytic activity in in vitro and in silico studies [10]. Since the factors involved in the pathogenesis of PF include chronic inflammation, an uncontrolled healing response, and progressive fibrosis or scarring, treatment with the systemic enzymes serrapeptase and nattokinase is a rational approach. Additionally, there is much anecdotal evidence that systemic enzyme supplementation decreases cough and breathlessness and reduces supplemental oxygen requirements in PF patients. However, evidence-based data are insufficient.

In chronic conditions, along with treating the disease and its symptoms, other aspects of patient care and wellbeing are important. However, current IPF care delivery models do not specifically address patient needs. As a result, patient advocacy bodies have called for action to develop a holistic approach to IPF patient care [11,12,13]. Patient-Reported Outcomes (PROs) are reported by patients and provide direct information about the perspective on their own health status and quality of life in the context of disease and its treatments [14]. PROs are dedicated tools to assess health and quality of life in patients [15,16]. These tools are multidimensional as they address various domains, such as symptoms, functional status, psychological, social and spiritual wellbeing. Targeted strategies to improve patient-centered care and to enhance quality of life and patient experience are crucial in progressive diseases such as IPF [17]. Besides IPF, there are several conditions of pulmonary fibrosis with identifiable causes, such as infections, environmental agents such as asbestos and silica, exposure to ionizing radiation, chronic conditions like lupus and rheumatoid arthritis, and certain medications. Shortness of breath, coughing, and decreased exercise tolerance are some common symptoms of all types of PF. Hence, this study aims to measure the effects of systemic enzyme supplementation on symptoms and quality of life in patients with PF using validated PROs.

## 2. Materials and Methods

### 2.1. Ethical Considerations

The present study was conducted as per the ethical principles contained in the current revision of the “Declaration of Helsinki 2013” and the harmonized integrated addendum to ICH E6(R1): Guidelines for Good Clinical Practice ICH E6(R2), and following all applicable laws and regulations. No vulnerable subject participated in the study. The study was approved by the Argus IRB, Tuscon, Arizona in October 2019.

### 2.2. Inclusion Criteria

Subjects who provided written informed consent, had a diagnosis of pulmonary fibrosis, and were aged ≥18 years were included in the study.

### 2.3. Exclusion Criteria

Subjects on any anti-coagulant medications, such as Aspirin, Plavix, Brillinta; subjects who had taken enzyme supplements within the 30 days immediately preceding the start of the study treatment; subjects with an allergy or sensitivity to enzyme supplements; subjects unable to read English, understand the questionnaires or follow study procedures; and subjects who were pregnant or planning to become pregnant were considered ineligible to participate in the study.

### 2.4. Study Design

This was a single-arm, open-label, proof-of-concept study examining the effects of the commercially available systemic enzyme supplements Serracor-NK + Serra Rx on symptoms and quality of life in patients with pulmonary fibrosis. The study was approved by the Argus IRB, Tuscon, Arizona. A total of 13 subjects were enrolled between November 2019 and August 2020. The subjects were recruited via advertising on the website and social media of the nonprofit Pulmonary Fibrosis NOW!. The subjects received the oral supplements Serracor-NK and Serra Rx three times a day for 12 weeks. The subjects completed three quality of life questionnaires: the World Health Organization-5 Well-Being Index (WHO-5), the University of California San Diego’s -Shortness of Breath Questionnaire (UCSD-SOB), and the Saint George’s Respiratory Questionnaire (SGRQ), at baseline and at various time points.

### 2.5. Screening and Enrolment

After providing informed consent, the subjects were screened, as mentioned in the study calendar (Table 1). After confirmation of their eligibility, the participants were enrolled in the study. All the subjects received the supplements and the assessments were performed as mentioned in the study calendar (Table 1). Day 100 was considered the end of the study period

### 2.6. Dose

Table 2 provides the dosing schedule for the supplements. The subjects received one capsule of Serracor-NK three times a day on days 1 to 4. They received two capsules of Serracor-NK three times a day on days 5 to 8. The subjects received a therapeutic dose of two capsules of Serracor-NK and one capsule of Serra Rx three times a day from days 9 to 92. The supplements were taken on an empty stomach (1 h before or 2 h after a meal) with a cup of water.

### 2.7. Statistical Analysis

The categorical variables were expressed as frequencies and percentages. The scoring and percentage of subjects showing improvement as compared to baseline using the study instruments were calculated as follows:

WHO (Five) Well-Being Index (WHO-5) [18]: The WHO-5 is a short self-reported measure of current mental well-being. It consists of five positively framed statements that subjects rate from 0 (none of the time) to 5 (all of the time). A raw score of 0 represents the worst possible quality of life (QOL) and 25 represents the best possible QOL. The percentage scores are calculated by multiplying the raw score by 4. A change in score of 10% is considered clinically significant.

The University of California San Diego -Shortness of breath questionnaire (UCSD-SOB) [19]: This is a 24 item measure that assesses self-reported shortness of breath while performing a variety of activities of daily living (ADL). The severity of SOB is rated from 0 (none at all) to 5 (maximum or unable to do because of shortness of breath) in 21 ADL. Three additional questions focus on fear of harm from overexertion, limitations, and fear caused by SOB. Scores range from 0 to 120, with higher scores indicating greater dyspnea. A change of 5 units is considered a reasonable minimal clinically important difference for this instrument.

Saint George’s Respiratory questionnaire (SGRQ) [20]: This is a standardized self-administered airways-disease-specific 50 item questionnaire split into three domains: symptoms (assessing the frequency and severity of respiratory symptoms), activity (assessing the effects of breathlessness on mobility and physical activity), and impact (assessing the psychosocial impact of the disease). Scores are weighted such that every domain score and the total score range from 0 to 100, as per the score calculation algorithms recommended by the questionnaire’s developer, with higher scores indicating more limitations [21]. A mean change score of 4 units is associated with slightly efficacious treatment, 8 units with moderately efficacious treatment and 12 units with very efficacious treatment.

## 3. Results

A total of 13 subjects completed the study. The median age range was 65–74 years, 69% were males vs. 31% females, 69% had a diagnosis of IPF, and 31% had a diagnosis of PF. The mean oxygen saturation (SpO2) was 94% (range, 88% to 99%); three patients had supplemental oxygen prescription. Forced Vital Capacity (FVC) ranged from 54% to 85% (data from nine subjects) and Diffusing Capacity of the Lung for Carbon monoxide (DLCO) predicted ranged from 40% to 90% (data from eight subjects). The end-of-study scores were compared to the baseline scores for the various instruments.

**Figure 1:**
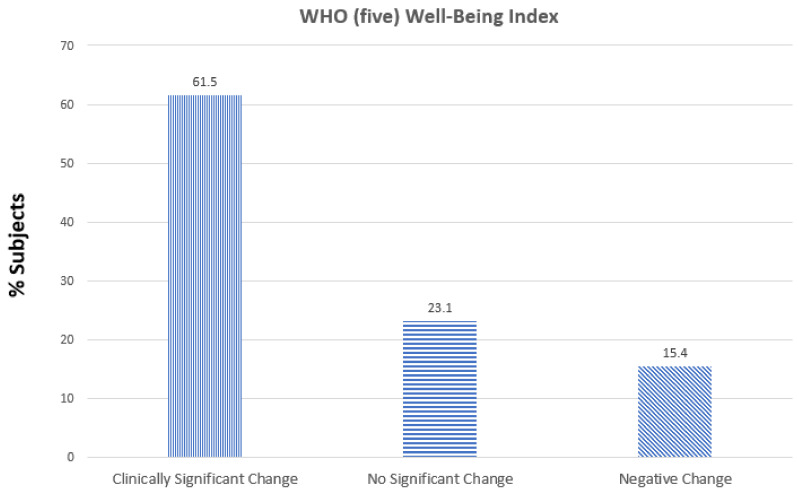
Effect of the supplements Serracor-NK and Serra Rx260 on WHO-(five) well-being index (1998 version) scores in subjects with pulmonary fibrosis (end-of-study scores as compared to baseline).

WHO (five) well-being index: Of the 13 subjects, 8 (61.5%) had a clinically significant positive change, 2 (15.4%) had no change and 2 (15.4%) showed a negative change in score as compared to baseline (Figure 1)

**Figure 2:**
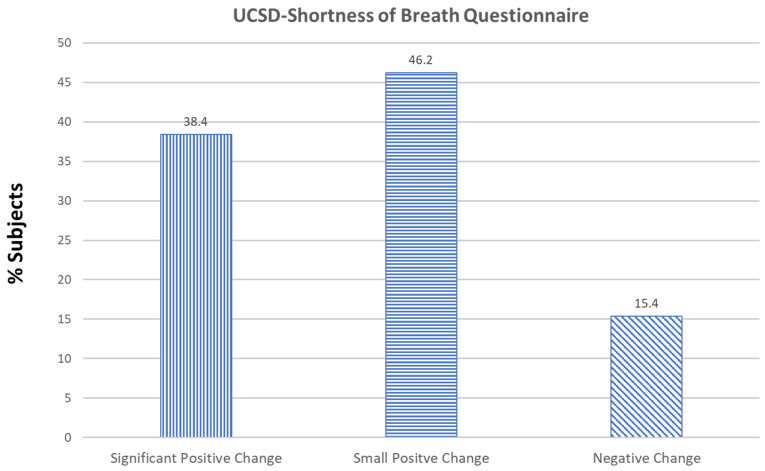
Effect of the supplements Serracor-NK and Serra Rx260 on University of California San Diego Shortness of Breath (UCSD-SOB) scores in subjects with pulmonary fibrosis (end-of-study scores as compared to baseline).

University of California San Diego Shortness of Breath (UCSD-SOB): Of the 13 subjects, 5 (38.4%) showed a positive significant change, 6 (46.2%) showed a small positive change that was not considered a reasonable MCID, and 2 (15.4%) showed a negative change in score as compared to baseline (Figure 2).

**Figure 3:**
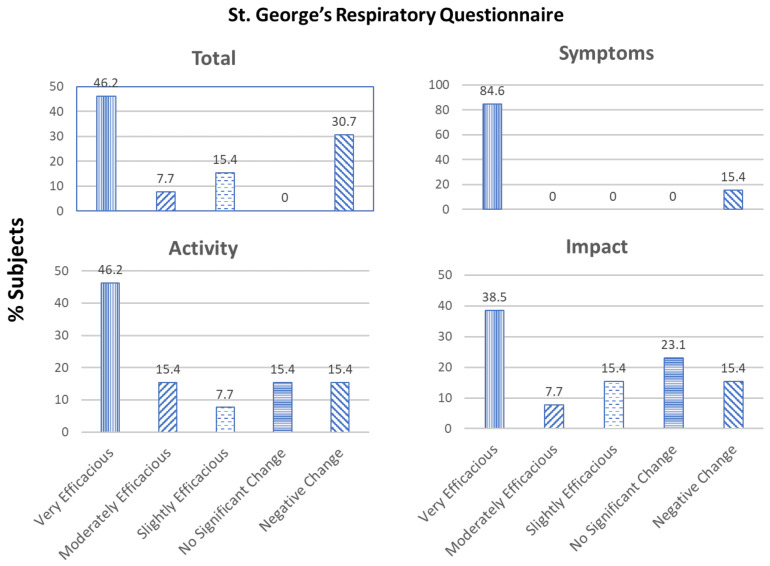
Effect of the supplements Serracor-NK and Serra Rx260 on St. George’s Respiratory Questionnaire (SGRQ): Total, Symptoms, Activity, and Impact scores of subjects with pulmonary fibrosis (end-of-study scores as compared to baseline).

St. George’s Respiratory Questionnaire (SGRQ): Of the 13 subjects, the treatment was found to be very efficacious in 6 (46.2%), moderately efficacious in 1 (7.7%), and slightly efficacious in 2 (15.4). Four (30.7%) subjects showed a decline in their total SGRQ scores. SGRQ scores were also calculated for the following three domains: Symptoms, Activity, and Impacts (Figure 3).

SGRQ-Symptoms: The treatment was found to be very efficacious in 11 (84.6%) subjects. Two (15.4%) subjects showed a decline in their SGRQ symptoms scores.

SGRQ-Activity: The treatment was found to be very efficacious in 6 (46.2%), moderately efficacious in 2 (15.4%), and slightly efficacious in 1 (7.7%) subject. No significant change was seen in 2 (15.4%) subjects and 2 (15.4%) subjects showed a decline in their SGRQ activity scores.

SGRQ-Impacts: The treatment was found to be very efficacious in 5 (38.5%), moderately efficacious in 1 (7.7%), and slightly efficacious in 2 (15.4%) subjects. No significant change was seen in 3 (23.1%) subjects and 2 (15.4%) subjects showed a decline in their SGRQ impacts scores.

## 4. Discussion

Health-Related Quality of Life (HRQOL) is a subjective and multidimensional concept that includes aspects of physical, mental, and social health. It focuses on the impact of health status on quality of life. Mental health problems can impact symptom control, exacerbation risk, and mortality. The WHO-5 is the most widely used questionnaire assessing subjective psychological wellbeing based on positive mood, vitality, and general interest in things. It has demonstrated validity both as a screening tool for depression and as an outcome measure in clinical trials [22]. We used this tool to measure the effectiveness of the intervention, since depression and anxiety are relatively common in patients with IPF and significantly influence quality of life in these patients [23]. Our data show that supplementation with systemic enzymes for 3 months helps to improve the feeling of wellbeing in PF patients.

The UCSD-SOBQ questionnaire is a relevant measure of the key symptoms of IPF [24]. It assesses dyspnea associated with specific activities of daily living (ADLs). The ability of this instrument to assess change in dyspnea over time in patients with IPF has been validated [25]. Subjects treated with a combination of systemic enzyme supplements for 3 months demonstrated improved scores on the UCSD-SOBQ as compared to baseline, suggesting that supplementation with Serracor-NK and Serra Rx reduces shortness of breath and improves patients’ ability to perform activities of daily living.

The SGRQ is a standardized disease-specific instrument designed to measure impact on overall health, daily life, and perceived well-being in patients with airways disease [21]. It has been designed to quantify changes in health following therapy [21]. Subjects treated with a combination of systemic enzyme supplements for 3 months demonstrated improved total, symptoms, activities, and impacts scores as compared to baseline, suggesting that treatment with these supplements decreases the frequency and severity of PF symptoms, improves patients’ ability to perform activities, and enhances social and psychological health,

In a progressive disease such as IPF, a small improvement in scores or even stable scores may indicate that the supplements are exerting beneficial effects. A small number of subjects showed no significant improvement or a worsening of scores as compared to baseline. It is possible that a longer period of supplementation may have been required in these subjects to produce benefits.

We chose a combination of Serracor-NK and Serra Rx based on historical, anecdotal, preclinical and clinical evidence on the beneficial effects of their ingredients [7,9,10,26]. Serracor-NK contains the fibrinolytic enzymes serrapeptase and nattokinase and other proteolytic enzymes, antioxidants, and essential vitamins and minerals, including bromelain, papain, lipase, rutin, amla, coenzyme Q10, and magnesium. Serra Rx is a serrapeptase supplement. The improvements observed in this study may be attributable to the fibrinolytic and inflammation-modulating effects of the two major systemic enzymes in the supplements, serrapeptase and nattokinase. A recent review details the available in vitro, in vivo and clinical evidence for the anti-inflammatory, analgesic, anti-edemic and fibrinolytic effects of serrapeptase [26]. Additionally, a study by et al. that investigated the effect of serrapeptase on sputum properties and symptoms in patients with chronic airway diseases showed that serrapeptase increased mucus clearance by reducing the viscoelasticity of sputum [27]. Nattokinase is a protease with strong fibrinolytic activity [28]. It has been used in Japan, Korea, and China for its anti-thrombotic and anti-coagulation effects. The improvement in the symptoms of dyspnea and coughing in our study and the resultant improvement in quality of life may be attributable to fibrin breakdown in the lungs and the easier clearing of mucus mediated by these systemic enzymes.

## 5. Conclusions

The present study suggests that the oral supplements Serracor-NK and Serra Rx improve symptoms, the ability to perform activities of daily living, and mental and physical well-being, thus enhancing overall quality of life in patients with pulmonary fibrosis. While there is no drug to cure IPF, these supplements can help to alleviate symptoms and improve quality of life in PF patients. This study was performed as a proof of concept. Further assessment in randomized, placebo-controlled trials with larger populations and for longer durations are warranted.

## Figures and Tables

**Table 1:** Study Calendar.

Required Study Procedure	Screening (Day-14 to Day-1)	Day 0 (Baseline/Registration)	Days from Registration			
			Day 36	Day 64	Day 92	Day 100
Informed Consent Form	√					
HIPAA Authorization	√					
Intake Form (demographics, disease severity, comorbidities)	√					
UCSD-SOB (approx. 7 minutes to complete)		√	√	√	√	
SGRQ (approx. 9 minutes to complete)		√	√	√	√	
WHO-5 (approx. 2 minutes to complete)		√	√	√	√	
Supplement Intake *			Daily, Starting day1			
Medication Log			Daily, Starting day1			
Follow-up						√

* Follow the dosing schedule (Table 2) for supplement intake.

**Table 2:** Dosing Schedule.

Directions: Take on an Empty Stomach (45 min before a Meal or 2 h after a Meal, with a Glass of Water)	
**Step 1** (Days 1–4)	**Take 1 capsule Settacor-NK, 3 times a day** Due to the body not being accustomed to systemic enzyme therapy, it is important to start at a minimal dosage and increase as needed. Minor symptoms of intestinal cleansing may occur.
**Step 2** (Days 5–8)	**Take 2 capsule Settacor-NK, 3 times a day** At this point, your body should be completed with the cleaning stage and becoming more accustomed to systemic enzyme therapy.
**Step 3** (Days 9–92)	**Take 2 capsule Settacor-NK + 1 capsule Serra Rx, 3 times a day** This is what we believe to be the therapeutic dosage.

## Data Availability

The conditions of our ethics approval do not permit public archiving of the data supporting the conclusions of the study. However, data described in the manuscript and code book will be made available upon request.
